# Cardiovascular 4D velocity mapping accelerated with k-t BLAST at 3.0 Tesla: 8-channel vs. 32-channel coil arrays

**DOI:** 10.1186/1532-429X-15-S1-O113

**Published:** 2013-01-30

**Authors:** Arshad Zaman, James Oliver, David M Higgins, Gerard Crelier, Sven Plein, John P Greenwood

**Affiliations:** 1 Cardiology, University of Leeds, Leeds, UK; 2 Philips Healthcare, Best, Netherlands; 3 University and ETH Zurich, Zurich, Switzerland

## Background

4D phase contrast CMR can be used to visualise and quantify cardiovascular flow and has become more widely available. However, the scan times tend to be long. Acceleration techniques such as SENSE can be useful. k-t BLAST is a recent method employed to reduce scan times even further. However, these newer acceleration techniques may potentially degrade image quality; this could be overcome by using a higher number of multi-coil arrays. 4D k-t BLAST velocity mapping has not been extensively used (or validated) for cardiovascular applications. The aim of this study was to evaluate the performance of 4D flow-sensitive CMR in the thoracic aorta with 8- and 32-channel coil arrays using k-t BLAST, compared to SENSE acceleration.

## Methods

Fifteen healthy subjects had their thoracic ascending aorta scanned on a 3.0T Philips Achieva TX system using: 1) 2D SENSE velocity mapping with 8 channels as the reference standard; 2) 4D-flow sequences accelerated with SENSE (SENSE 1.6, 25 phases, Venc 200, 20 slices, resp. nav.) and k-t BLAST (k-t factor 5, training matrix 11, 25 phases, Venc 200, 20 slices), using both 8 and 32 channels.

Data processing, quantification, and visualisation were performed using GT Flow (V2.02, Gyrotools, Zurich) and compared to the results from 2D-flow acquisitions. Contours were drawn in the ascending aorta at the level of the pulmonary artery (at peak systole). Quality of the magnitude images and pathline visualisation was evaluated independently by two cardiologists using a scale from 1 (poor) to 4 (excellent). The maximum/minimum score possible for the 15 subjects was 60/15 for the magnitude and pathline data (n=15), the mean of these was then calculated (mean±SD).

## Results

**Figure 1:**
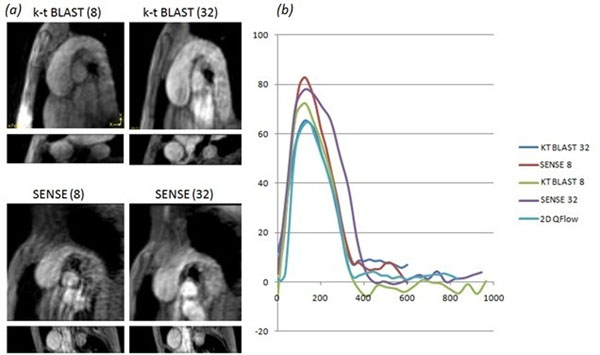
a) 4D k-t BLAST and SENSE accelerated magnitude images (using 8 and 32 channels), k-t has improved image quality b) Flow curve analysis revealing very good agreement between the 4D k-t BLAST (32 channel) and the standard 2D SENSE.

Mean scan times for the 4D SENSE navigated scans were 25.2 minutes (acquisition time 4.2 minutes), whereas for the k-t acquisitions scan mean times were 5.5 minutes (Table 1). Data acquisition with 32 channels consumed a large proportion of the scanner hard-disk space, which could cause significant workflow problems. However, acquisition using 32 channels produced data with significantly (P<0.05) higher image quality scores compared with that with 8 channels (Fig. 1). 4D k-t BLAST utilising 32 channels (mean image quality score 42.2 ± 3.6) provided higher quality visualisation of pathlines when compared with 8 channels (30.2 ± 4.2) or 4D SENSE 32ch and 8ch (22 ± 6.3; 16.3 ± 7.4).

**Table 1:** 4D flow pulse sequence parameters

Sequence	Nav	Acquisition time (mins)	Filesize on database (GBs)	Analysis time (mins)	Image quality
4D SENSE (8)	Y	4.2 (25.2 nav)	0.4	40	16.3 ± 7.4

4D SENSE (32)	Y	4.2 (25.2 nav)	1.1	40	22 ± 6.3

4D K-t BLAST (8)	N	5.5	0.2	40	30.2 ± 4.2

4D K-t BLAST (32)	N	5.5	0.7	40	42.2 ± 3.6

2D SENSE	N	8	0.06	40	14.3 ± 7.4

Quantitative flow (Figure 1) and Bland-Altman analysis revealed very good agreement (with no significant bias) between the 4D k-t BLAST (64.3 ml/s ±5.3) and 2D SENSE (63.7 ml/s ±7.1) flow.

## Conclusions

4D flow-sensitive MRI of the ascending thoracic aorta using k-t BLAST and 32 channel coils allows a reduction in total scan time whilst improving overall image quality compared to a standard 2D SENSE and 4D SENSE acquisitions. The use of 32 channels rather than 8 channels with the 4D k-t BLAST was also preferable in terms of image quality.

## Funding

AZ funded by the Flexibility and Susceptibility Funding stream of West Yorkshire CLRN. SP funded by a British Heart Foundation Fellowship.

